# Hippocampal neurons with stable excitatory connectivity become part of neuronal representations

**DOI:** 10.1371/journal.pbio.3000928

**Published:** 2020-11-03

**Authors:** Tim P. Castello-Waldow, Ghabiba Weston, Alessandro F. Ulivi, Alireza Chenani, Yonatan Loewenstein, Alon Chen, Alessio Attardo

**Affiliations:** 1 Max Planck Institute of Psychiatry, Munich, Germany; 2 Graduate School of Systemic Neurosciences Ludwig-Maximilians-Universität, Munich, Germany; 3 The Hebrew University, Jerusalem, Israel; 4 Weizmann Institute of Science, Rehovot, Israel; Institute of Science and Technology Austria, AUSTRIA

## Abstract

Experiences are represented in the brain by patterns of neuronal activity. Ensembles of neurons representing experience undergo activity-dependent plasticity and are important for learning and recall. They are thus considered cellular engrams of memory. Yet, the cellular events that bias neurons to become part of a neuronal representation are largely unknown. In rodents, turnover of structural connectivity has been proposed to underlie the turnover of neuronal representations and also to be a cellular mechanism defining the time duration for which memories are stored in the hippocampus. If these hypotheses are true, structural dynamics of connectivity should be involved in the formation of neuronal representations and concurrently important for learning and recall. To tackle these questions, we used deep-brain 2-photon (2P) time-lapse imaging in transgenic mice in which neurons expressing the Immediate Early Gene (IEG) *Arc* (activity-regulated cytoskeleton-associated protein) could be permanently labeled during a specific time window. This enabled us to investigate the dynamics of excitatory synaptic connectivity—using dendritic spines as proxies—of hippocampal CA1 (cornu ammonis 1) pyramidal neurons (PNs) becoming part of neuronal representations exploiting *Arc* as an indicator of being part of neuronal representations. We discovered that neurons that will prospectively express *Arc* have slower turnover of synaptic connectivity, thus suggesting that synaptic stability prior to experience can bias neurons to become part of representations or possibly engrams. We also found a negative correlation between stability of structural synaptic connectivity and the ability to recall features of a hippocampal-dependent memory, which suggests that faster structural turnover in hippocampal CA1 might be functional for memory.

## Abstract 2

The cellular events that bias neurons to become part of neuronal representations and engrams are largely unknown. This study of the dynamics of excitatory synaptic connectivity of CA1 hippocampal neurons expressing the Immediate Early Gene Arc reveals that synaptic stability can bias neurons to become part of representations and that faster structural turnover in dorsal hippocampal CA1 might be functional for memory.

## Introduction

Patterns of neuronal activity represent experiences in the brain and are important not only for real-time computations but also for long-term memory formation. In fact, it has been possible to identify ensembles of neurons undergoing coordinated activity and activity-dependent plasticity by the expression of Immediate Early Genes (IEGs), tagging them using opto- or chemogenetics, and manipulating their activity to achieve control over memory recall [1–10]. Thus, ensembles of neurons undergoing coordinated activity-dependent plasticity are believed to form a long-term cellular trace of memory or a cellular engram [11–14].

The level of excitability plays an important role in biasing pyramidal neurons (PNs) to become active and eventually to become involved in engrams [10,15–22]. Although the transcription factor CREB (cAMP response element-binding protein) has been identified as a key factor in biasing neuronal excitability and recruitment into engrams [10,15–18], the cellular events that prime PNs to undergo activity-dependent plasticity and the cellular changes these neurons undergo upon experience are largely unknown.

In the hippocampus, higher turnover of structural synaptic connectivity [19,20] might underlie the turnover of ensembles of neurons representing experience and undergoing activity-dependent plasticity [21–25]. However, the relationship between turnover of structural synaptic connectivity and the probability of participating in a neuronal representation is still unexplored. If turnover of structural synaptic connectivity underlies turnover of neuronal representations, it might have a direct relationship with hippocampal learning and recall as observed in the neocortex [26–29]. However, the relationship between hippocampal structural synaptic dynamics and hippocampal-dependent learning and recall has not been established.

To confront these fundamental questions, we used deep-brain 2-photon (2P) time-lapse imaging [30] to track dendritic spine dynamics—as proxies for structural excitatory synaptic dynamics—in transgenic mice in which neurons expressing the IEG activity-regulated cytoskeleton-associated protein (*Arc*) could be permanently labeled during a specific time window [31]. To address the limitations of 2P imaging in resolving all spines in hippocampal cornu ammonis 1 (CA1) [19], we performed internally controlled experiments by comparing spine dynamics of (i) hippocampal CA1 PNs expressing and not expressing *Arc* and (ii) of the same PNs before and after *Arc* expression, all within the same individuals. In addition, as a control, we tracked dendritic spine dynamics of PNs that expressed the same fluorescent tag as neurons expressing *Arc* but in a non-activity–dependent fashion.

We could thus investigate the influence of structural excitatory synaptic plasticity on neuronal representations or engrams and the link between synaptic plasticity and hippocampal-dependent memory recall in a trace fear conditioning (TFC) learning task.

## Results

### Using the promotor of the IEG *Arc* to label hippocampal CA1 neurons representing experience

The promotor of the IEG *Arc* has been previously used to label neurons taking part in representations of experience and in memory engrams [8,23,32]. Thus, we used the Arc-Cre^ERT2^; Ai9 double knock-in mouse line [31], in which the promoter of *Arc* drives expression of a Cre recombinase fused to the estrogen receptor triple mutant 2 (ERT2) domain, leading to tamoxifen (TAM)-dependent expression of tdTomato (tandem dimer Tomato) during a defined time window in neurons active during exploration of an enriched environment (EE, S1A Fig).

**Fig 1:**
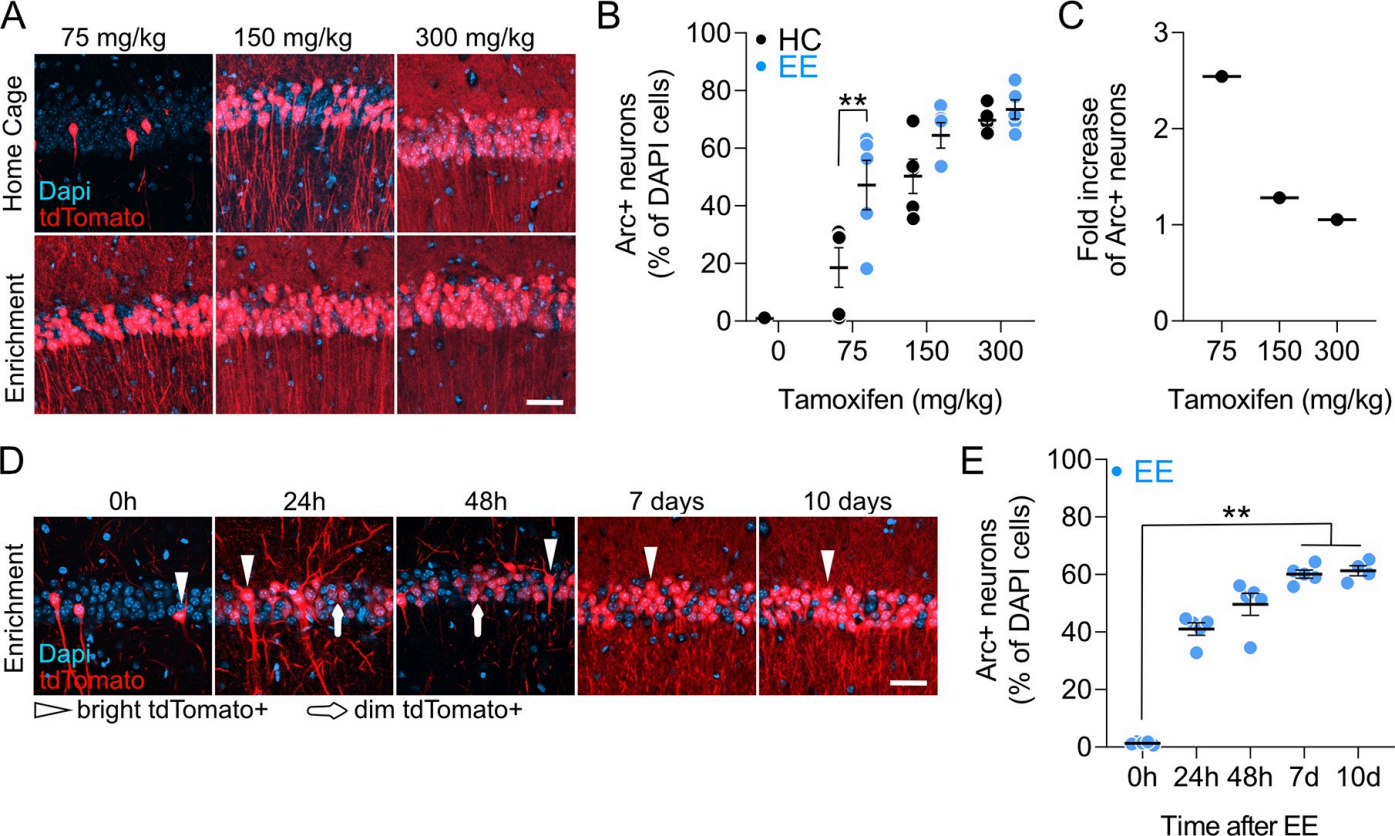
Characterization of TAM-dependent tdTomato expression. (A) Confocal images of the dorsal CA1 of Arc-Cre^ERT2^; Ai9 animals after permanence in HC (upper row) or spending 2 h in an EE (lower row). Mice received either 75 mg/kg, 150 mg/kg, or 300 mg/kg TAM and were killed 10 d after injection. Single z-plane (5-μm z-step, 8–10 focal planes). Scale bar, 100 μm. (B) Proportion of ArcTom+ neurons after HC (black circles) or EE (blue circles) per mouse (**p = 0.0044; *N* = 5 mice per group; 2-way ANOVA with Šidák’s multiple comparisons correction). Error bars are SEM. (C) A 75-mg/kg TAM injection led to a 2.5-fold increase of ArcTom+ neurons after EE compared to HC. (D) Confocal images of the dorsal CA1 of Arc-Cre^ERT2^; Ai9 animals killed at different time points after 16 h in EE. Mice received 75 mg/kg TAM and were killed 0 h, 24 h, 48 h, 7 d, or 10 d after the end of EE exploration. Single z-plane (5-μm z-step, 8–10 focal planes). Scale bar, 100 μm. (E) Proportion of ArcTom+ neurons at different time points after EE per mouse (**p_0–7_ = 0.0029, **p_0–10_ = 0.0018; *N* = 5 mice per group: Kruskal–Wallis test with Dunn's multiple comparisons correction). Error bars are SEM. All the data of this figure can be found in the S1 Data file. *Arc*, activity-regulated cytoplasmic-associated protein; ArcTom, Arc-tdTomato; CA1, cornu ammonis 1; EE, enriched environment; ERT2, estrogen receptor triple mutant 2; HC, home cage; TAM, tamoxifen; tdTomato, tandem dimer Tomato.

To investigate the specificity of Arc-Cre^ERT2^; Ai9 labeling, we compared the amount of labeling of the dorsal CA1 in mice housed in their home cage (HC) to mice allowed to explore an EE. We injected groups of Arc-Cre^ERT2^; Ai9 mice with increasing doses of TAM (Fig 1A). For each dose, 1 group explored an EE for 2 h while the other group remained in its HC. A single 75-mg/kg TAM injection labeled, on average, 50% of neurons upon exploration of EE and 20% of neurons during HC, thus attaining a 2.5-fold increase in the number of Arc-tdTomato (ArcTom)+ neurons over baseline (Fig 1B and 1C). Although higher doses of TAM led to progressively higher labeling in both EE and HC conditions, labeling between EE and HC reached significance only at 75 mg/kg (Fig 1B; S1 Table for complete list of *p*-values and statistical tests). We thus used a single 75-mg/kg TAM injection in Arc-Cre^ERT2^; Ai9 mice to label Arc-expressing CA1 PNs during exploration of an EE.

To investigate the kinetics of Arc-Cre^ERT2^; Ai9 labeling, we injected groups of Arc-Cre^ERT2^; Ai9 mice with a single 75-mg/kg dose of TAM prior to exploration of an EE and analyzed the time course of appearance of tdTomato fluorescence in the dorsal hippocampal CA1 (Fig 1D). Although we detected very few tdTomato + cell immediately after EE (Fig 1D, 0 h), tdTomato expression visibly increased 24 h after EE, and it reached significance at the population level 7 and 10 d after EE (Fig 1E). Interestingly, during earlier time points (24 h and 48 h), the majority of tdTomato+ neurons (24 h: 97.47% ± 1.48%; 48 h: 97.44% ± 1.21%) displayed fluorescence levels approximately one order of magnitude lower than at later time points (7 d and 10 d), indicating de novo protein expression early after EE reaching saturation 1 week after EE exposure.

Finally, we were also able to label neurons active during training of the hippocampus-dependent learning task TFC (S1B–S1D Fig). The average number of tdTomato+ neurons labeled after TFC was lower than after exploration of EE (approximately 25% versus 50%), possibly reflecting the shorter duration of this task, in addition to the difference in sensory complexity between the 2 tasks.

### Retrospective and prospective tracking of dendritic spines in CA1 PNs expressing and not expressing *Arc*

**Fig 2:**
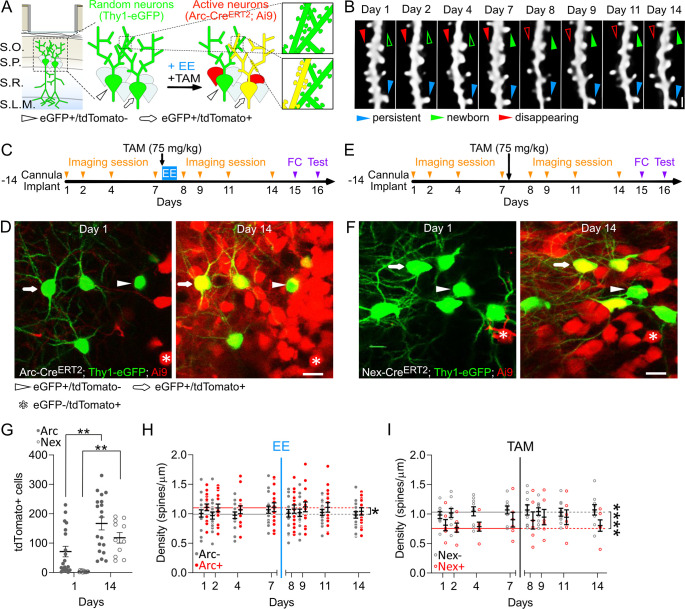
Retrospective and prospective tracking of dendritic spines in CA1 PNs participating and not participating to representations. (A) Schematic description of the preparation and of the experiment’s rationale. Green, eGFP+ neurons; red, tdTomato+ neurons; yellow, eGFP/tdTomato double+ neurons. (B) In vivo 2P time lapse of the same dendritic branch and spines over 14 d. MIPs of up to 15 z-planes (Z-step, 1 μm). Scale bar, 1 μm. (C) Experimental timeline. (D) 2P images (MIPs of up to 5 z-planes, 2-μm z-step) of the dorsal CA1 of Arc-Cre^ERT2^; Ai9; Thy1-eGFP live mice. Scale bar, 20 μm. (E) Nex-Cre^ERT2^; Ai9; Thy1-eGFP; mice underwent the same experimental procedure as in A and B but were never exposed to EE. (F) 2P images (MIPs of up to 5 z-planes, 2 μm z-step) of the dorsal CA1 of Nex-Cre^ERT2^; Ai9; Thy1-eGFP live mice. Scale bar, 20 μm. (G) Number of CA1 PNs expressing tdTomato before (day 1) and after the administration of TAM in Arc-Cre^ERT2^; Ai9; Thy1-eGFP and Nex-Cre^ERT2^; Ai9; Thy1-eGFP animals (**p = 0.0012 and **p = 0.0036; N_Arc_ = 19, *N*_Nex_ = 11 imaged regions; 2-way ANOVA with Šidák’s multiple comparisons correction). (H, I) Spine densities of (H) ArcTom+, ArcTom−, and (I) NexTom+ and NexTom− neurons are significantly different from each other (*p = 0.0218 and ****p < 0.0001; Mann–Whitney U test; *N*_ArcTom−_ = 136, *N*_ArcTom+_ = 128, *N*_NexTom−_ = 72, *N*_NexTom+_ = 48) but do not change with time (*p* > 0.2; 1-sample Wilcoxon test median spine density days 1–7 versus distribution of mean density per mouse on each day; *N*_ArcTom−_ = 17, *N*_ArcTom+_ = 16, *N*_NexTom−_ = 9, *N*_NexTom+_ = 6). Data points are mean density per neuron. Red: prospective or actual tdTomato+ neurons, gray: prospective or actual tdTomato− neurons, solid circles: Arc-Cre^ERT2^; Ai9; Thy1-eGFP mice, empty circles: Nex-Cre^ERT2^; Ai9 Thy1-eGFP mice, black bars: means ± SEM, horizontal lines: median baseline densities per each group. Blue vertical line, time point of EE + TAM injection; gray vertical line, time point of TAM injection. All the data of this figure can be found in the S1 Data file. Arc, activity-regulated cytoplasmic-associated protein; ArcTom, Arc-tdTomato; CA1, cornu ammonis 1; EE, enriched environment; eGFP, enhanced green fluorescent protein; ERT2, estrogen receptor triple mutant 2; FC, fear conditioning; MIP, maximum intensity projection; Nex, neuronal helix-loop-helix protein; NexTom, Next-tdTomato; PN, pyramidal neuron; SLM, Stratum Lacunosum-Molecolare; SO, Stratum Oriens; SP, Stratum Pyramidale; SR, Stratum Radiatum; TAM, tamoxifen; tdTomato, tandem dimer Tomato; Thy1, thymocyte differentiation antigen 1; 2P, 2-photon.

To visualize dendritic spines in CA1 neurons expressing *Arc* during exploration of an EE, we obtained triple transgenic Arc-Cre^ERT2^; Ai9; Thy1-eGFP (thymocyte differentiation antigen 1-enhanced green fluorescent protein) mice. In these mice, 1% to 5% of all CA1 PNs expressed cytoplasmic eGFP [33], and an independent and overlapping PN subpopulation expressed *Arc*-driven activity-dependent tdTomato. We used deep-brain 2P optical imaging to track dendritic spines in the dorsal CA1 of these mice to longitudinally investigate the structural postsynaptic dynamics of PNs expressing and not expressing *Arc* within the same subjects (Fig 2A and 2B).

We imaged the dendritic spines of basal dendrites of CA1 PNs in 7 Arc-Cre^ERT2^; Ai9; Thy1-eGFP mice for 1 week (S2 Table for number of neurons and dendrites imaged). On the evening of day 7, we injected TAM and moved the mice to an EE. We returned the mice to their HC on the morning of day 8 and imaged them for 7 more days (Fig 2C). 56.8% of the eGFP+ PNs we tracked in live animals showed *Arc*-driven tdTomato expression (Fig 2D, arrows). We excluded any neurons expressing tdTomato since the beginning of imaging from our analysis (Fig 2D, asterisks).

To control for potential effects of repeated imaging, TAM injection, tdTomato expression, and scoring variability, we performed the same experiment but without exposure to EE on neuronal helix-loop-helix protein (Nex)-Cre^ERT2^; Ai9; Thy1-eGFP transgenic mice (Fig 2E). In these mice, the Cre^ERT2^ recombinase is expressed in a subset of CA1 PNs independent of neuronal activity [34]. In fact, when we compared the numbers of tdTomato+ cells in the CA1 of Arc-Cre^ERT2^; Ai9; Thy1-eGFP and of Nex-Cre^ERT2^; Ai9; Thy1-eGFP mice that had been housed in the HC or exposed to an EE, we found that EE led to an increased number of tdTomato+ cells only in Arc-Cre^ERT2^; Ai9; Thy1-eGFP mice, but not in Nex-Cre^ERT2^; Ai9; Thy1-eGFP mice (S1E and S1F Fig).

Approximately 52% of the eGFP+ PNs we tracked in live animals became tdTomato+ (Fig 2F, arrows); we will refer to these as Nex-tdTomato (NexTom)+ neurons. We excluded any neurons expressing tdTomato since the beginning of imaging (Fig 2F, asterisks).

When we compared the numbers of tdTomato+ cells in the CA1 of Arc-Cre^ERT2^; Ai9; Thy1-eGFP mice prior to and after exposure to EE and of Nex-Cre^ERT2^; Ai9; Thy1-eGFP mice prior to and after TAM injection, we found an increase in the number of tdTomato+ cells in both groups and no difference between mice exposed to EE (Arc-Cre^ERT2^; Ai9; Thy1-eGFP) and mice that stayed in their HC (Nex-Cre^ERT2^; Ai9; Thy1-eGFP) (Fig 2G).

Dendritic spine densities of prospective ArcTom+ and ArcTom− neurons and of NexTom+ and NexTom− neurons differed during baseline but were stable after TAM injection with or without exposure to EE (Fig 2H and 2I). These data demonstrate that repeated imaging, TAM injection, and exposure to EE do not grossly affect the number of dendritic spines.

The orders of the dendrites of ArcTom+ and ArcTom− neurons we analyzed were comparable to each other, and likewise for NexTom+ and NexTom− neurons (S1G Fig).

### Prospective *Arc*-expressing CA1 PNs display higher structural postsynaptic stability

Average stability of postsynaptic structural connectivity can arise from slower or faster temporal dynamics of dendritic spines. Neurons with slower dynamics of spines (i.e., small gains and small losses) are more stably connected with the same presynaptic partners, than neurons with faster dynamics (i.e., high gains and high losses). To investigate the strength of connectivity of active neurons, we analyzed the temporal dynamics of the gain and loss of dendritic spines.

**Fig 3:**
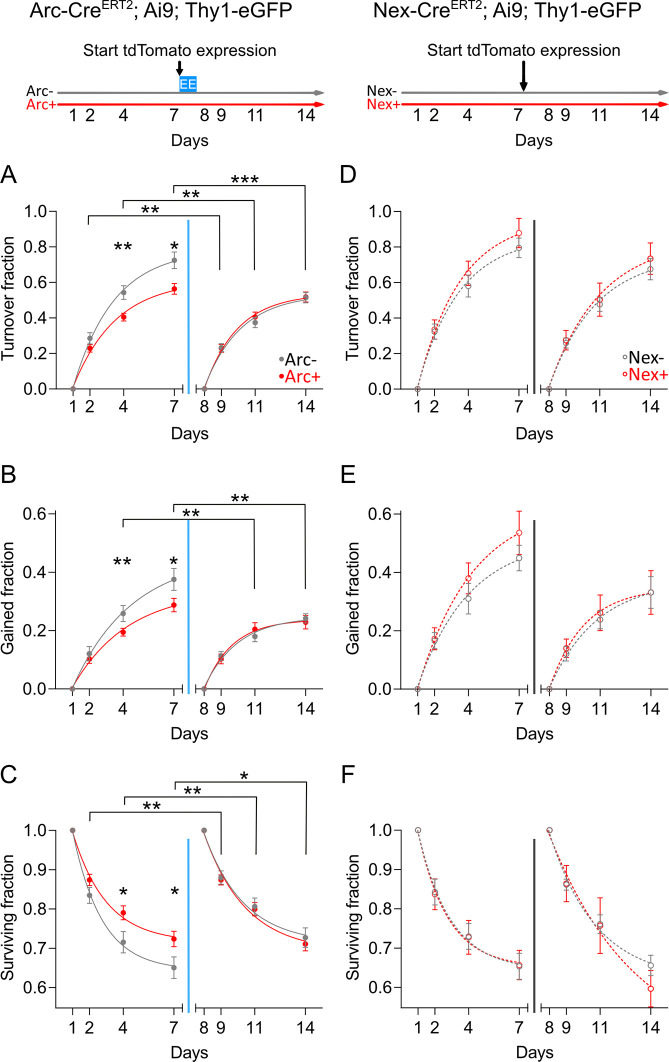
CA1 PNs that will become part of a representation display higher structural postsynaptic stability. (A, B) The turnover fraction (A) and the fraction of dendritic spines gained (B) of prospective ArcTom+ neurons was lower than prospective ArcTom− neurons prior to EE (*p = 0.015, **p = 0.008 and *p = 0.02, **p = 0.009; permutation test; *N* = 10^5^ permutations). The turnover and gain of ArcTom−, but not ArcTom+, neurons decreased after EE (**p = 0.003, **p = 0.002, ***p = 0.0008 and **p = 0.002, **p = 0.004; paired Wilcoxon rank–sum test; *N*_ArcTom−_ = 17, *N*_ArcTom+_ = 16). (C) The survival fraction of prospective ArcTom+ neurons was higher than prospective ArcTom− neurons prior to EE (*p = 0.02 and *p = 0.03; permutation test; *N* = 10^5^ permutations). Survival of dendritic spines of ArcTom−, but not ArcTom+, neurons increased after EE (*p = 0.025, **p = 0.0099, and **p = 0.004; paired Wilcoxon rank–sum test; *N*_ArcTom−_ = 17, *N*_ArcTom+_ = 16). (D–F) The turnover fraction (D), the fraction of dendritic spines gained (E), and the survival fraction (F) of NexTom− and NexTom+ neurons were not different during baseline (*p* > 0.2; permutation test; *N* = 10^5^ permutations) and were not affected by TAM injection (*p* > 0.05; paired Wilcoxon rank–sum test; *N*_NexTom−_ = 9, *N*_NexTom+_ = 6). (A–F) Data points are the number of spines that turned over (A, D), were gained (B, E), or survived (C, F), normalized to day 1 or 8. Red: prospective or actual td-Tomato+ neurons, gray: prospective or actual tdTomato− neurons. Solid circles: Arc-Cre^ERT2^; Ai9; Thy1-eGFP mice, empty circles: Nex-Cre^ERT2^; Ai9; Thy1-eGFP mice. Curves: single exponential fits to the data, blue vertical line: time point of EE exposure + TAM injection; gray vertical line: time point of TAM injection. Error bars are the SD of the distribution of surrogate spine turnover, gain, or loss obtained by 2-level bootstrapping (level 1 neurons, level 2 spines); see Materials and methods for details. All the data of this figure can be found in the S1 Data file. Arc, activity-regulated cytoplasmic-associated protein; ArcTom, Arc-tdTomato; ArcTom, Arc-tdTomato; CA1, cornu ammonis 1; EE, enriched environment; eGFP, enhanced green fluorescent protein; ERT2, estrogen receptor triple mutant 2; Nex, neuronal helix-loop-helix protein; NexTom, Nex-tdTomato; PN, pyramidal neuron; TAM, tamoxifen; tdTomato, tandem dimer Tomato; Thy1, thymocyte differentiation antigen 1.

Prior to EE, prospective ArcTom+ neurons displayed a lower turnover rate than ArcTom− neurons (Fig 3A). Lower turnover resulted from lower gain and higher survival rates (Fig 3B and 3C). After EE, gain rate decreased whereas survival rate increased in ArcTom− neurons, leading to a decreased turnover rate and ultimately no statistical difference between ArcTom+ and ArcTom− neurons (Fig 3A, 3B and 3C). Comparing turnover, gain, and loss pooled over the entire baseline and after EE periods produced similar results (S2A–S2C Fig). Spine turnover, gain, and loss rates did not show any difference between NexTom+ and NexTom− neurons (Fig 3D–1F and S2D–S2F Fig).

Changes in brightness between imaging sessions or motion artifacts might result in apparent variations in size of spines and dendrites; spine size differences might bias detectability and lead to apparent differences in gain and survival. We thus quantified the variation in the size of stable spines as well as the variation in the diameter of the dendritic shafts of ArcTom− and ArcTom+ neurons through time. We found no difference between the sizes of ArcTom− and ArcTom+ spines or dendritic shafts and no variation through time (S2G and S2H Fig), thus excluding the possibility that changes in spine gain and loss rates could be due to increase in apparent spine size.

In summary, our data show that *Arc*-expressing PNs display higher excitatory postsynaptic structural synaptic stability even before their activation, and exposure to a new environment increases the structural stability of PNs that do not express *Arc*.

### Synaptic sites with recurrent dendritic spines are more stably connected in prospective *Arc*-expressing CA1 PNs

**Fig 4:**
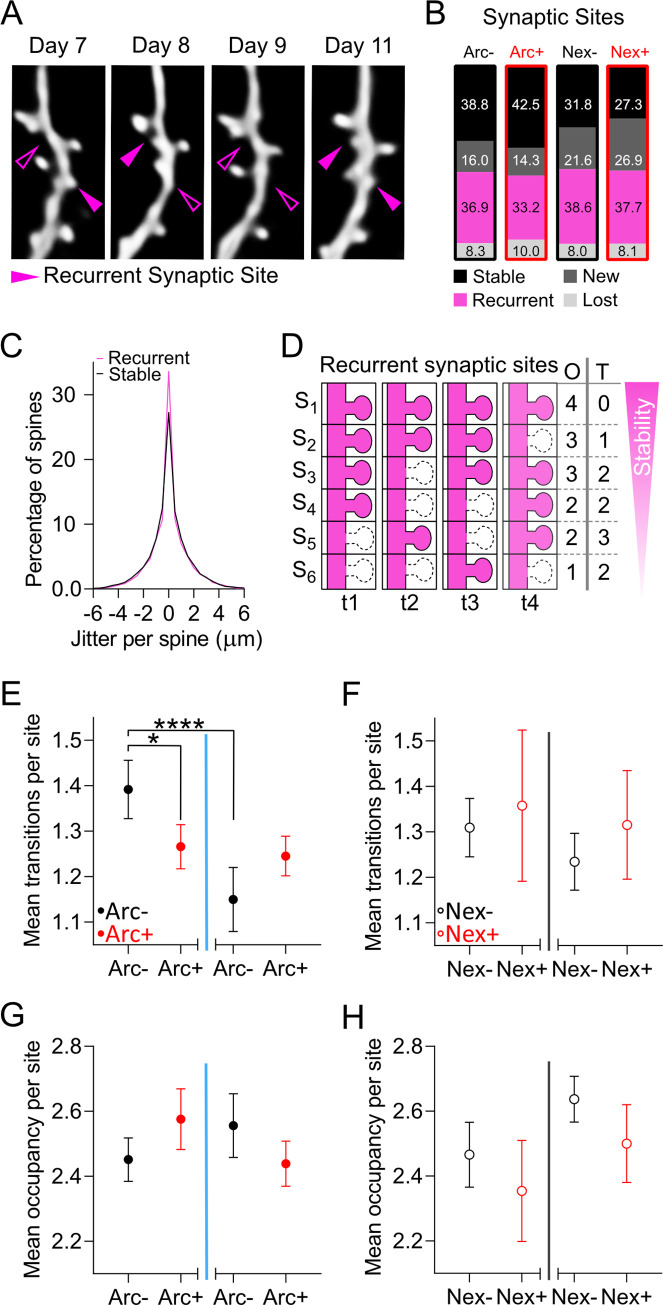
Recurrent dendritic spines are more stable in CA1 PNs that will become part of a representation. (A) In vivo 2P time lapse of the same dendritic branch and spines over 5 d. MIPs of up to 7 z-planes (Z-step, 1 μm). Scale bar, 1 μm. (B) Percentages of stable, new, recurrent, and lost synaptic sites defined over the 14 imaging days. (C) The positional jitters for stable (black) and recurrent (magenta) dendritic spines were not different (*p* = 0.6; Mann–Whitney U test; *N*_Stable_ = 15,176, *N*_Recurrent_ = 8,444). (D) Schematic description of recurrent synaptic sites (s) at consecutive time points displaying different numbers of time points (t) occupied (O) and transitions between occupied and not occupied states (T). (E) There were fewer transitions between occupied and not occupied states of recurrent sites in ArcTom+ compared to ArcTom− neurons (*p = 0.014; permutation test; *N* = 10^5^ permutations). Transitions decreased upon exposure to EE in ArcTom− neurons (****p < 0.0001; paired Wilcoxon rank–sum test; *N*_ArcTom−_ = 17). (F) The number of transitions between occupied and not occupied states of recurrent sites of NexTom− and NexTom+ neurons were not different during baseline (*p* > 0.03; permutation test; *N* = 10^5^ permutations) and were not affected by TAM injection (*p* > 0.01; paired Wilcoxon rank–sum test; *N*_NexTom−_ = 9, *N*_NexTom+_ = 6). (G, H) Occupancy of recurrent sites of ArcTom−, ArcTom+, NexTom−, and NexTom+ neurons were not different during baseline (*p* > 0.05; permutation test; *N* = 10^5^ permutations) and were not affected by exposure to EE or TAM injection (*p* > 0.01; paired Wilcoxon rank–sum test; *N*_ArcTom−_ = 17, *N*_ArcTom+_ = 16, *N*_NexTom−_ = 9, *N*_NexTom+_ = 6). (E–H) Data points are the mean number of transitions between occupied and not occupied states (E, F) or the mean number of time points occupied (G, H) during baseline after exposure to EE or TAM injection of ArcTom− (solid circles, black), ArcTom+ (solid circles, red), NexTom− (open circles, black), and NexTom+ (open circles, red) neurons. Error bars are the SD of the distribution of surrogate spine turnover, gain, or loss obtained by 2-level bootstrapping (level 1 neurons, level 2 spines); see Materials and methods for details. All the data of this figure can be found in the S1 Data file. Arc, activity-regulated cytoplasmic-associated protein; ArcTom, Arc-tdTomato; CA1, cornu ammonis 1; EE, enriched environment; MIP, maximum intensity projection; Nex, neuronal helix-loop-helix protein; NexTom, Nex-tdTomato; PN, pyramidal neuron; TAM, tamoxifen; tdTomato, tandem dimer Tomato; 2P, 2-photon.

Approximately 35% of dendritic spines disappeared and recurred at a site along the dendrite whose location was indistinguishable from the position in which they were first detected (Fig 4A and 4B). The positional jitter of spines recurring on these sites, or recurrent spines, was indistinguishable from the jitter of stable spines (Fig 4C), and synaptic sites with recurrent spines were occupied more often than expected by chance (S3A and S3B Fig).

Given the sparseness and the geometry of CA3 to CA1 connectivity [35,36], recurrent spines are more likely to connect the same pre- and postsynaptic neurons than nonrecurrent spines. We thus investigated the stability of connectivity of synaptic sites with recurrent dendritic spines by quantifying the number of transitions between presence and absence of the corresponding spine (Transitions, T) and the number of time points in which each site was occupied by a spine (Occupancy, O) (Fig 4D and S3C–S3J Fig). Synaptic sites with recurrent dendritic spines showed fewer Transitions in ArcTom+ than in ArcTom− neurons before EE and exposure to EE decreased the number of Transitions of ArcTom− neurons (Fig 4E). These differences were absent in Nex-Cre^ERT2^; Ai9; Thy1-eGFP mice (Fig 4F). We found no significant differences in Occupancy (Fig 4G and 4H).

These data show that, similarly to other spines, recurrent dendritic spines tend to be more stable in *Arc*-expressing CA1 PNs.

### Baseline density and survival of dendritic spines of CA1 PNs negatively correlate with contextual, but not tone, freezing during recall of a TFC learning task

**Fig 5:**
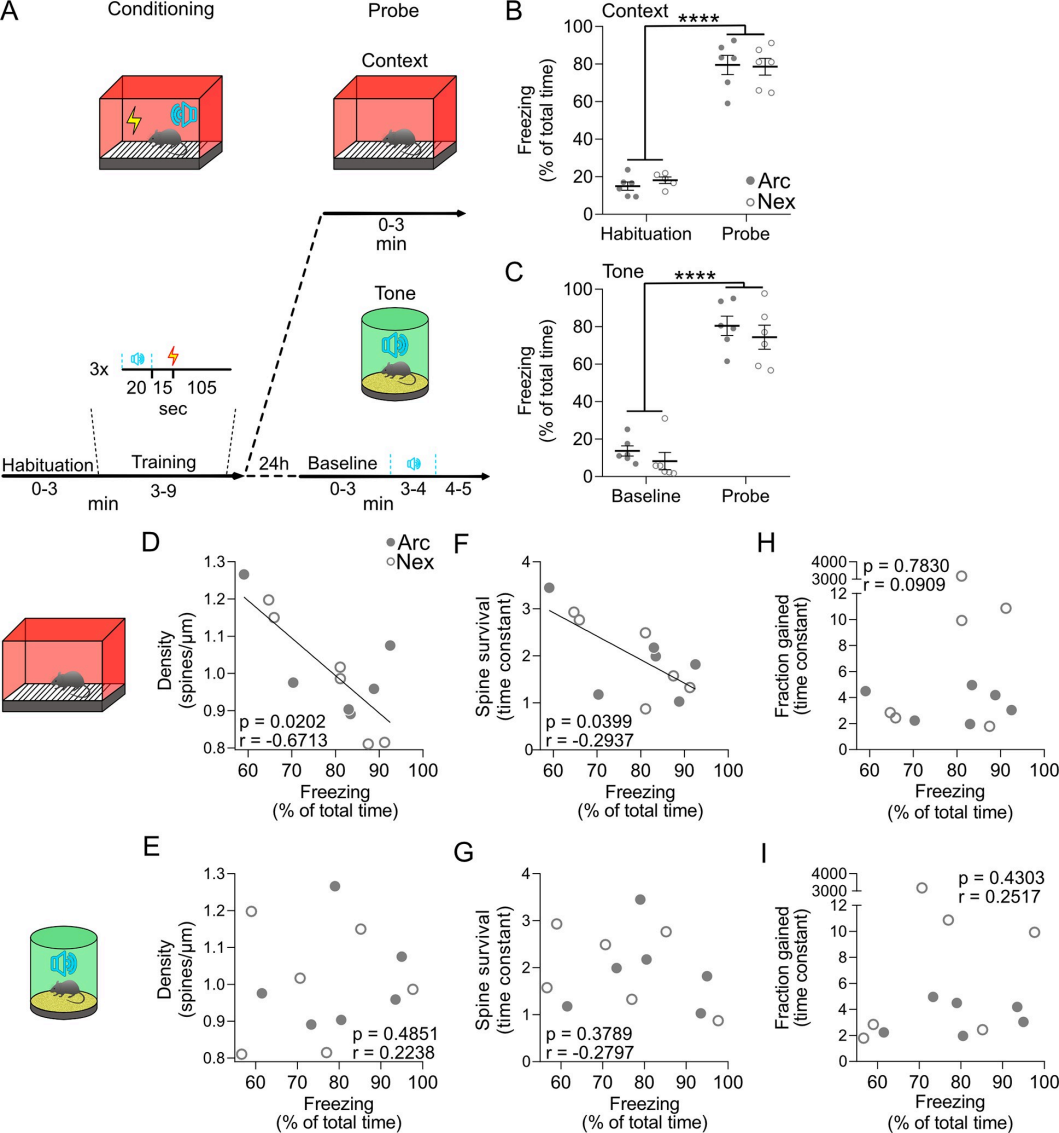
Density and survival of dendritic spines of CA1 PNs negatively correlate with hippocampal-dependent memory. (A) Schematic description of the TFC learning task. (B, C) Arc-Cre^ERT2^; Ai9; Thy1-eGFP (Arc, solid circles) and Nex-Cre^ERT2^; Ai9; Thy1-eGFP (Nex, open circles) mice displayed increased freezing during the context (B) and tone (C) probe trials (****p < 0.0001; *N*_Arc_ = 6, *N*_Nex_ = 6; 2-way ANOVA with Tukey's multiple comparisons correction). Circles are percentage freezing for each mouse during 3 min habituation (day 15) and 3 min context probe (day 16, B) or during 3-min baseline (day 16) and 4-min tone probe (day 16, C); horizontal bars: means ± SEM. (D, F, H) Baseline densities (D) and time constants of the survival (F), but not gain (H), rates of spines of Arc-Cre^ERT2^; Ai9; Thy1-eGFP (solid circles) and Nex-Cre^ERT2^; Ai9; Thy1-eGFP (open circles) significantly correlated to freezing to the context (*p = 0.02, *p = 0.03, *p* = 0.8, and R = −0.67, R = −0.29, R = 0.09; Spearman correlations; *N* = 12). (E, G, I) Baseline densities (E) and time constants of the survival (G) and gain (I) rates of spines of Arc-Cre^ERT2^; Ai9; Thy1-eGFP (solid circles) and Nex-Cre^ERT2^; Ai9; Thy1-eGFP (open circles) did not significantly correlate to freezing to the tone (*p* = 0.48, *p* = 0.37, *p* = 0.8 and R = 0.22, R = −0.27, R = 0.25; Spearman correlations; *N* = 12). All the data of this figure can be found in the S1 Data file. Arc, activity-regulated cytoplasmic-associated protein; CA1, cornu ammonis 1; eGFP, enhanced green fluorescent protein; Nex, neuronal helix-loop-helix protein; PN, pyramidal neuron; TFC, trace fear conditioning; Thy1, thymocyte differentiation antigen 1.

Next, we wanted to investigate the relationship between dendritic spine dynamics and memory recall in a hippocampal-dependent learning task. Immediately after the last day of in vivo imaging, we trained the animals in a TFC memory task (day 15) and monitored their freezing to the context and to the tone separately on the next day (day 16, Fig 2C and 2E and Fig 5A). Arc-Cre^ERT2^; Ai9; Thy1-eGFP and Nex-Cre^ERT2^; Ai9; Thy1-eGFP mice showed significant freezing to the context and to the tone alone and were statistically indistinguishable (Fig 5B and 5C). These results suggest that both groups learned the association of the context and tone to the shock and that exploration of an EE for 16 h (Arc-Cre^ERT2^; Ai9; Thy1-eGFP mice only) did not significantly affect the recall in this task.

To investigate the relationship between baseline spine dynamics and memory recall, we pooled the data from Arc-Cre^ERT2^; Ai9; Thy1-eGFP and Nex-Cre^ERT2^; Ai9; Thy1-eGFP mice. These mouse lines are congenic and do not display any phenotypic difference; furthermore, all animals had identical experience prior to EE exposure. Average density and survival rates of spines of all neurons (tdTomato+ and tdTomato−) per mouse during the baseline period (before the EE or TAM) predicted the level of freezing to the context but failed to predict the level of freezing to the tone alone (Fig 5D–5G). Average gain rates per mouse did not predict the level of freezing to the context or the tone (Fig 5H and 5I).

When we analyzed the correlations between dendritic spine dynamics of animals that underwent EE exploration (Arc-Cre^ERT2^; Ai9; Thy1-eGFP mice) or remained in their HC (Nex-Cre^ERT2^; Ai9; Thy1-eGFP mice), we found that the average density, the survival, and the gain of spines did not predict freezing (S4A–S4L Fig).

Altogether, these data show a linear relationship between baseline dendritic spine dynamics of CA1 PNs and some of the features of hippocampal-dependent memory recall.

## Discussion

### Labeling specificity and dendritic spine tracking of CA1 PNs participating in representations

The promotor of the IEG *Arc* can label neurons taking part in representations of experience and in memory engrams [8,23,32]. Because we did not test directly whether activation or inactivation of *Arc*-expressing cells would lead to changes in memory encoding or recall, in this work we consider *Arc*-driven tdTomato expression to label CA1 neurons taking part in the neuronal representation (in brief representation neurons) of a novel environment. Using a single 75-mg/kg TAM injection, we detected a 30% increase in CA1 ArcTom+ cells after EE exposure compared to remaining in the HC (Fig 1B) and a similar percent increase after TFC (S1C Fig). These results are consistent with previous work reporting increased *Arc* transcription or immunoreactivity upon FC or exposure to an EE [37,38]. In addition, approximately 10% to 20% of cells were also labeled in the HC (Fig 1B and S1C Fig), which might reflect the biological variability of *Arc* expression under baseline conditions or leakiness of the labeling system. These baseline labeling levels, however, suggest that some of the ArcTom+ neurons whose dendritic spines we tracked in vivo after EE might not be specific for the representation of the EE. This, in turn, means that our work underestimates the differences in dynamics between neurons representing EE and neurons not representing EE. Increasing doses of TAM led to higher recombination and a decrease in the difference between EE and HC conditions, which might result from the combination of leakiness and increased bioavailability of TAM (because of higher doses), both of which were previously described [31].

2P imaging is widely used to track neuronal structure [39], but it cannot resolve all spines in the hippocampal CA1 [19], which might lead to conflicting results [19,20,40]. To address this issue and to control for various potential other sources of noise, we performed within-subject internally controlled experiments and tracked dendritic spine dynamics of neurons expressing the same fluorescent tag as active neurons but in a non-activity–dependent fashion. Thus, although our measure of the synaptic stability of neurons is not absolute, we were able to uncover significant differences in synaptic stability.

### Higher structural postsynaptic connectivity of CA1 PNs participating in representations

Dendritic spine densities of prospective ArcTom+ and ArcTom− neurons and of NexTom+ and NexTom− neurons differed during baseline. Because these differences are constant through time, exposure to EE, injection of TAM, and expression of tdTomato (Fig 2H and 2I), we assume that they reflect stochastic differences between subpopulations of neurons.

A key finding of our work is that despite substantial stationary spine density, CA1 PNs that will become part of a representation show higher stability of dendritic spines in their basal dendrites. We cannot tell whether higher postsynaptic structural stability corresponds to higher functional synaptic connectivity. However, assuming no difference in the proportion of dendritic spines carrying active synapses between prospective representation and nonrepresentation neurons, higher stability of dendritic spines translates to longer durations of time in which representation neurons are connected with their excitatory presynaptic partners. Especially in a brain region with high synaptic turnover such as hippocampal CA1 [19,20], a bias towards synaptic stability will likely result in increased functional connectivity that, in turn, could render a representation neuron more excitable and thus receptive to memory allocation [10,15–18,41–45]. Moreover, increased synaptic transmission could lead to local plasticity [46,47] and prime CA1 representation neurons to become more active [48,49]. Further experiments should clarify the extent to which high structural stability leads to increased neuronal excitability.

### Exploration of an EE stabilizes the connectivity of neurons that do not participate in the representation

We did not detect a significant change in spine density in the dorsal CA1 after an exposure to EE for 16 h, in contrast to others who reported density increase in the neocortex and hippocampus [50–52] or a decrease in CA1 PNs expressing *Arc* [37] upon EE. This discrepancy might be due to the different duration of the EE, to the time after EE at which spine density was quantified, or to the statistical fluctuations between different groups of animals.

We found stabilization of structural connectivity upon exploration of a novel EE, which is in line with previous studies in the neocortex [26,27,29]. However, although these studies could not identify representation neurons, we found that stabilization of connectivity occurred predominantly in CA1 neurons that were not part of the representation of the EE. One possible explanation for this finding is that neurons that will prospectively participate in representations have already reached a structural stability ceiling and thus cannot be stabilized any further, whereas neurons that will prospectively not participate in representations might respond to factors secreted during EE. In fact, the protein Arc could itself mediate the transfer of mRNAs to be translated in neighboring neurons, thus possibly informing inactive neurons of the status of their neighbors [53]. In addition, it has recently been shown that it is more difficult to induce LTP (long-term potentiation) between highly structurally connected CA3 and CA1 engram neurons [54], which supports a ceiling effect for increase in functional connectivity between highly structurally connected neurons. Stabilizing the connectivity of neurons not involved in the current representation might prime these neurons to take part in a future representation, thus competing with neurons that are part of the current representation. This would support turnover of neurons involved in representations at different time points. Whereas orthogonalization of the neuronal populations taking part of CA1 representations at different time points has been demonstrated at similar timescales [21,23], there are also forces that push for similarity between representations of events close in time [55,56]. It would be important to probe the extent to which artificial stabilization of synaptic connectivity of single neurons can influence these neurons to become part of a future representation.

### Higher density and survival of CA1 dendritic spines during baseline predict lower freezing to the context, but not to the tone

The density and the survival of hippocampal dendritic spines predicted some features of hippocampal-dependent recall, such as the amount of freezing to the context, but failed to predict others, such as the amount of freezing to the sound alone. This is consistent with the idea that different features of the association of context, tone, and shock are represented or encoded in different subregions of hippocampal CA1. For example, it has been demonstrated that different information streams are spatially segregated in the dorsal hippocampal CA1 [57–62]. Thus, our data indicate that the basal dendrites of CA1 might be involved in encoding the context—as suggested by the association of their synaptic dynamics with freezing to the context—but only marginally involved in the encoding of the tone. Future experiments should clarify whether changes in spine dynamics in the basal aspect of the dorsal CA1 are causally linked to levels of freezing to the context.

The association between dynamics of dendritic spines in the hippocampus and freezing in an hippocampus-dependent memory task suggests that similarly to the neocortex [26–29], structural synaptic dynamics in the hippocampus can support cognitive functions. Interestingly, animals that displayed higher density or stability of CA1 dendritic spines during baseline showed lower levels of freezing, analogous to results from the retrosplenial cortex [63]. This might seem counterintuitive based on the notion of neocortical dendritic spines being the cellular correlates of permanently recorded information. However, in contrast to the neocortex, the hippocampus does not store long-term information, but rather provides a large synaptic space to quickly represent new information. This information is held for a limited amount of time as it progressively transits to neocortical long-term storage sites [64,65]. In this framework, fast structural turnover of synapses [19,20] might endow hippocampal CA1 neurons with a faster reset rate of the synaptic space to allow for new information to be encoded. This might be particularly relevant in the CA1, where the density of dendritic spines in PNs is relatively high and seems to be stable over time [19,20,66], creating conditions under which it might be more difficult to generate new synaptic space purely by adding new dendritic spines. In these conditions, the amount of synaptic space available for new information should be mostly a function of the rate at which space can be made available by clearing spines representing older information. Thus, faster structural turnover—i.e., shorter survival time—could make synaptic space available faster, which might benefit encoding and result in better recall.

In summary, our results suggest that lower synaptic stability at the population level coexists with higher synaptic stability of a subpopulation of representation—or possibly engram—neurons. We think of these being the cellular substrates for two opposing but complementary forces enabling learning in the hippocampus; whereas high synaptic turnover at the population level increases the rate at which synaptic space is made available to encode new information, higher synaptic stability of a subpopulation of neurons selects the neurons tasked with encoding of this information temporarily.

## Materials and methods

Further information and requests for reagents may be directed to and will be fulfilled by the corresponding author. All published reagents can be shared on an unrestricted basis. All the data the figures refer to can be found in the S1 Data file. All original raw data will be made available by the corresponding author on request.

### Ethics statement

All animal procedures conformed to the guidelines of the Max Planck Society and the local animal authority (Regierung von Oberbayern–Veterinärwesen) and were approved in the license (Tierversuchslizenz) ROB-55.2Vet-2532.Vet_02-16-48.

### Experimental model and subject details

Mice were held on a 12-h light/dark cycle. Experiments were conducted during the 12-h light period. Littermates were group-housed with a maximum of 5 mice per cage, with access to food and water ad libitum. All experimental procedures were conducted with 12-week–old male and female mice.

### Labeling of neurons active during exploration of EE or TFC

Mice received a single intraperitoneal injection of TAM (75 mg/kg, 150 mg/kg, or 300 mg/kg of body weight) right before being placed into the EE. Tamoxifen was dissolved in 5% of the final volume in 100% ethanol and further diluted with corn oil to a final concentration of 10 mg/ml. The solution was heated up to 37°C before injection. After exposure to the EE (2 h or 16 h), mice were transferred back into their HC. Enriched environments were created by connecting 2 rat-cages (37 cm × 60 cm) with an acrylic tunnel (20 cm × 15 cm), resulting in a total area of 4,440 cm^2^. Enriched cages contained tunnels, wooden climbing sticks, wooden shelters, running wheels, seesaws, cotton pads, hair curlers, wooden blocks, swinging hammocks, and toys that mice could open and that contained food pellets. Cages also contained a second level connected to the ground floor by a wooden ladder and consisting of a wooden board and climbing ropes, allowing mice to reach the lid grit. Food was hidden in the bedding material and spread around the arena to encourage mice to explore the environment.

### Histology

We perfused mice intracardially with 1× phosphate-buffered saline (PBS) containing heparin, followed by 4% paraformaldehyde (PFA) in PBS. We then dissected the brains and placed them in 4% PFA in 1× PBS for 24 h. Brains were then transferred to 30% sucrose in PBS for 48 h. Brain slices (50 μm thick) were prepared with a vibratome (Thermo Fisher Scientific Microm HM 650V; Waltham, MA, USA). Slices were quenched with 150 mM glycine in ddH_2_0 for 15 min and permeabilized with 0.2% Triton X-100 in PBS for 1 h. Slices were incubated with DAPI (1:1,000 diluted in PBS; Thermo Fisher Scientific) for 5 min, washed with PBS, and mounted onto slides with mounting medium (Vectashield; Vector Laboratories, Burlingame, CA, USA).

### Quantification of tdTomato+ cells

To quantify the proportion of Arc- or Nex-tdTomato+ CA1 PNs ex vivo, we used a confocal microscope (Zeiss LSM 800; Carl Zeiss, Oberkochen, Germany) and acquired image stacks (319.28-μm^2^ single section area, 5-μm z-step, 8–10 focal planes) of 4 representative fields in the dorsal CA1 per mouse using a 40× objective (Zeiss Plan-Apochromat 40x/1.4 Oil DIC [UV] VIS-IR). We acquired DAPI fluorescence (405-nm excitation and 465-nm emission wavelengths) to identify neuronal nuclei and tdTomato fluorescence (561-nm excitation and 618-nm emission wavelengths) to identify Arc- or Nex-tdTomato+ cells. We then manually counted DAPI+, tdTomato+, and double-positive neurons using the plugin Cell Counter (ImageJ). When the same neuron was visible in more than 1 z-slice, we only counted it once in the z-plane in which the diameter of the soma was the largest.

To quantify the number of Arc- or Nex-tdTomato+ CA1 PNs in vivo, we used a 2P microscope (Bruker Ultima IV; Billerica, MA, USA) and acquired image stacks (239.26-μm^2^ single section area, 2-μm z-step, 41–65 focal planes) of 1–4 representative fields in the dorsal CA1 per mouse using a 25× objective (Olympus XLPlan N 25×/1.00 SVMP; Olympus, Tokyo, Japan). We acquired tdTomato fluorescence (1,040-nm excitation and 618-nm emission wavelengths) to identify tdTomato+ cells. We then manually counted tdTomato+ cells on day 1 before and 7 days after TAM injection on day 14 using the plugin Cell Counter (ImageJ).

### Implantation of the imaging cannula

We prepared imaging cannulas as described in [30]. To induce anesthesia, mice were put into an anesthesia induction chamber that was flooded with 2.5% isoflurane in pure O_2_. During the surgery, the anesthesia was held by 1.5% isoflurane in pure O_2_ directly delivered to the mouse’ nostrils. The appropriate depth of anesthesia was confirmed by the absence of the toe pinch reflex. Meloxicam (1 mg/kg) and Vetalgin (200 mg/kg) were administered by subcutaneous injection, and ophthalmic ointment (Bepanthen cream) was applied to protect the eyes. Nonrupture ear bars (David Kopf Instruments, Tujunga, CA, USA) were positioned to stabilize the skull. After hair removal, the skin was disinfected, the skull—roughly from the frontal to the interparietal bone—was exposed, and a drop of lidocaine (approximately 10 mg) was applied to the skull. A small craniotomy of the left frontal bone was performed using a microdrill with 0.5-mm burr. A 0.86-diameter stainless-steel screw was inserted and fixed by the application of Metabond. A 3-mm–diameter craniotomy centered at 2 mm mediolateral and −2.3 mm anteroposterior from bregma was performed using a trephine drill, the dura was removed, and the cortical matter was slowly ablated using a blunt needle connected to a vacuum pump until reaching the fibers of the corpus callosum. The imaging cannula was inserted into the craniotomy, applying slight pressure onto the tissue to stabilize the preparation, and the cannula was fixed and sealed to the skull using Metabond (Parkell, Edgewood, NY, USA). A custom head plate was positioned and fixed with dental cement. The following 2 days, mice received meloxicam (1 mg/kg) once per day for postoperative analgesia.

### In vivo 2P imaging of dendritic spines

Two to three weeks after implantation of the imaging cannula, mice were anesthetized with 2.5% isoflurane and placed under the microscope (Bruker Ultima IV) onto a 37°C heating pad (CMA 450) while the head was fixed via a head plate holder. During imaging, mice were kept under constant anesthesia (1.5% isoflurane). To manually align the imaging cannula to the light path, we used a 4× objective (Olympus Plan N 4×/0.10) and fluorescent light (X-Cite 120Q) to visualize the top and the bottom of the cannula. We adjusted the 3 separate rotational degrees of freedom of the animal’s head independently to align the axis of the cannula to the axis of imaging. We considered the cannula aligned when the top and bottom circular ends of the cannula were concentric. For 2P imaging, the objective was changed to a 25× water immersion objective (Olympus XLPlan N 25×/1.00 SVMP). To excite eGFP and tdTomato, we used a pulsed infrared laser tuned to 920 nm and 1,040 nm, respectively. On days 1 and 14, overview images of the eGFP and the tdTomato channel were acquired consecutively with 1×, 2×, and 5× digital zoom to relocate the dendrite of interest and to identify double-positive neurons. To image dendritic spines, we acquired z-stacks (48.18-μm^2^ single section area, 1-μm z-step, 5–60 z-steps, zoom 10×, 28.6- to 115.5-mW laser power at the sample) of the dendrite of interest using a resonant scanner at each time point. We acquired each z-plane 4 times before moving to the next plane.

### Image postprocessing

To compensate for motion artifacts, we split each of the stacks into 4 single stacks containing a single acquisition per each x-plane. We then registered each stack in the x-dimension by using the ImageJ plugins Turboreg and Stackreg (Philippe Thévenaz, GitHub) and deconvolved each stack separately using blind deconvolution (AutoquantX3). Finally, we registered the 4 corresponding z-planes from each stack and averaged them using ImageJ. We aligned the resulting average stack once more through the z-plane.

### Scoring of dendritic spines and identification of dendritic order

Dendritic spines were manually scored using a custom graphical user interface written in MATLAB (The MathWorks, Natick, MA, USA) that concatenated all time points in a loop and hid the date on which each time point was acquired; thus, the operator was blind to the experimental condition while counting. First, we traced dendrites in 3 dimensions with a node-connected line that permitted measuring the length of the dendritic segment. Then, we marked a spine in 1 time point and moved to the next time point to identify the same spine. Initially, we marked 5 spines per dendrite present in all 8 time points, which were used as stable fiduciary points for rigid-body registration across time. After registration, we counted all spines in each dendritic segment. Spines that disappeared were scored as lost spines on that day, and spines that were not present at the previous imaging time point were counted as new spines. New spines that appeared at a previously lost position were considered spines in a recurrent location.

Two-P overview image stacks were used to identify if a dendrite belonged to a tdTomato+ neuron by manually tracing each dendritic segment back to its soma and to establish the dendritic order. Each basal dendrite originating from the soma was classified as a first-order dendrite. After the first branching point, both emerging dendrites were allocated as a second-order dendrite, independent of the size or the diameter of the 2 dendrites. Using this technique, the most distal identified dendritic segments belonged to the sixth order.

### Metrics used to quantify spine dynamics and size

In S2 Fig, fractional gain and fractional loss were defined as the number of spines gained or lost between each time point and the next one, normalized by the number of spines present in the first time point. Turnover rate was defined as the sum of the fractional gain and fractional loss. All values during baseline or after EE/TAM were pooled together and treated as a single distribution. To quantify the size of a spine, we drew manually a region of interest (ROI) around a spine in a single z-plane. We then calculated the sum of the fluorescence of all pixels included in the ROI of the spine—integrated density of fluorescence (IDF)—using ImageJ. The IDF thus accounted for the size of the ROI and the fluorescence of the ROI. The IDF of the spine was normalized to the IDF of a fixed-sized ROI of the dendritic shaft of the same z-plane below the spine.

In Fig 3, fractional gain and fractional survival were defined as the number of spines gained or surviving between the first and each of the other time points, normalized by the number of spines present in the first time point (Baseline) or after EE/TAM. Fractional turnover rate was defined as a sum of the fractional gain and fractional loss (1 − surviving fraction).

In Fig 4, the jitter of a spines’ location over time was defined as the distribution of the differences between the position of that spine on the linearized dendrite at the first time point it appeared and the positions of the same spine at all other time points in which it was detected. Recurrent sites were defined over the whole duration of the experiment (8 time points) and were characterized by a spine present at least at 1 time point, then lost and then reappearing at least once in a location that was visually indistinguishable from the first location it was detected in. Occupancy (O) was defined as the number of time points the recurrent site contained a spine. Transitions (T) were defined as the number of switches from presence to absence or absence to presence of a spine in a recurrent site.

In Fig 5 and S4 Fig, the density of spines per mouse was calculated as the average of the densities of the dendrites of that mouse. To calculate the time constant τ of the turnover, gain, and surviving rates per mouse, we fitted exponential growth to plateau (for turnover and the growth) or single exponential decay (for survival) curves to the distributions of fractional turnover, gain, or survival of the dendrites of that mouse.

### TFC

On the training day (day 1 or 15), mice were put into a square conditioning chamber (19 cm × 19 cm, black metal walls, stainless-steel grid floor, white light illumination, and ethanol odor; Panlab, Barcelona, Spain), which we defined as Context A. Following 3 min of habituation, mice received 3 pairings of a tone (80 dB, 9 kHz, 20 s duration, CS) and a mild electric foot shock (0.75 mA, 1 s duration, US), with a trace of 15 s between the tone and the shock and an intratrial interval of 105 s. On probe day (day 5 or 16), mice were tested for their memory recall. To test context memory recall, we placed mice into Context A for 3 min. Thirty min later, to test tone memory recall, mice were placed into a novel context (a round chamber of 15-cm diameter, transparent acrylic walls, bedding, white light illumination, and acetic acid odor), which we defined as Context B. Mice explored Context B for 4 min, after which we administered the CS for 1 min. During all exposures to Context A and B, the position of the mouse was tracked automatically, and the freezing response was recorded and quantified in real time with ANY-maze (Stoelting, Wood Dale, IL, USA). The amount of freezing was calculated as the percentage of total exploration time during which the mice were immobile. Immobility for more than 250 ms was scored as freezing.

### Quantification and statistical analysis

For statistics, we used the Mann–Whitney test (2-tailed), 1-sample Wilcoxon test, paired Wilcoxon, 2-way ANOVA, and Spearman correlation. For post hoc analysis for multiple comparisons, we used Šidák’s, Dunn’s, Bonferroni, or Tukey’s test. Statistical analysis and plotting were done with Prism 8 (GraphPad) software. *p ≤ 0.05, **p ≤ 0.01, ***p ≤ 0.001, ****p < 0.0001.

In S3 Fig, to establish whether recurrent sites were occupied more often than expected by chance, we calculated the probability to observe any given occupancy (including the measured occupancy) using a hypergeometric function:

P(X=k)=(Mk)(N−Mn−k)(Nn),

where *p* = the probability of having *k* recurrent sites occupied, *N* = potential sites that can be occupied, *M* = recurrent sites available, *n* = sites that can be occupied on a given day, and *k* = recurrent sites occupied on a given day. The measured occupancy was significantly different from chance if the sum of all probabilities bigger than the probability observed for the data was greater than 0.95 (probability mass function).

$$0.05>p\;value=1-\sum_{b>P}a_{b},$$

with *b* = control variable, *p* = probability, and *a*_*b*_ = function of control variable.

In Figs 3 and 4 and S3 Fig, to quantify the confidence intervals in the measured fractions of spine gain, spine loss, spine turnover, occupancies, and flips, we used a 2-level bootstrapping method, which accounts both for heterogeneity between neurons and for heterogeneity between spines within a neuron. In short, for each group of neurons (ArcTom+, ArcTom−, NexTom+, and NexTom−), we randomly sampled (with replacement) the corresponding number of neurons from that group. For each of these sampled neurons, we randomly sampled (with replacement) the corresponding number of spines or sites. Then, we used these sampled spines to compute surrogate spine gain, spine loss, spine turnover, occupancies, and flips. This procedure was repeated 10,000 times, and the error bars denote the standard deviations of the distribution of these quantities across the different samples. To test the significance of the difference between tdTomato+ and tdTomato− groups, we used a permutation test (10^5^ permutations) on the identities of the neurons (ArcTom+ versus ArcTom− or NexTom+ versus NexTom−). To test the significance of a change following EE (ArcTom+ before EE versus ArcTom+ after EE, etc.), we used a paired test; we measured, for each neuron, the difference between the measure (fractions of spine gain, spine loss, spine turnover, occupancies, and flips) before and after the EE and used a 2-sided Wilcoxon rank–sum test to compute the significance of the change.

## Supporting information

S1 Fig **(A) Picture showing a representative EE.** (B) Confocal images of the dorsal CA1 of Arc-Cre^ERT2^; Ai9 animals after permanence in HC (up) or after a TFC training. Mice received 75 mg/kg TAM. Single z-plane (5-μm z-step, 8–10 focal planes). Scale bar, 100 μm (C) The proportion of ArcTom+ neurons (solid orange circles) after TFC was significantly higher than the proportion of ArcTom+ neurons (solid black circles) after HC. *p* = 0.0167; Mann–Whitney U test; *N*_HC_ = 3, *N*_TFC_ = 6. Horizontal bars: means ± SEM. (D) Arc-Cre^ERT2^; Ai9 mice displayed increased freezing during the context probe trial (***p < 0.0006; Mann–Whitney U test; *N* = 7). Circles are percentage freezing for each mouse during 3 min exposure to the context during training and 3 min exposure to the context during probe. Horizontal bars: means ± SEM. (E) Confocal images of the dorsal CA1 of Nex-Cre^ERT2^; Ai9 animals after 2 h permanence in HC (up) or in EE (low). Mice received 75 mg/kg TAM. Single z-plane (5-μm z-step, 8–10 focal planes). Scale bar, 100 μm (F) The proportion of NexTom+ (empty circles) after 75-mg/kg TAM injection was not different between exposure to HC (black) and EE (blue) but different from ArcTom+ neurons (solid circles). *p* = 0.84; Mann–Whitney U test; *N* = 5 mice per group. Error bars are SEM. (G) Schematic definition of the dendritic order of the imaged dendrites and proportion of dendrites in each class per group. All the data of this figure can be found in the S1 Data file. Arc, activity-regulated cytoplasmic-associated protein, ArcTom, Arc-tdTomato; CA1, cornu ammonis 1; EE, enriched environment; ERT2, estrogen receptor triple mutant 2; HC, home cage; Nex, neuronal helix-loop-helix protein; NexTom, Nex-tdTomato; TAM, tamoxifen; tdTomato, tandem dimer Tomato.
(TIF)

S2 Fig Comparisons of fractional turnover, gain, and loss per epoch. (A) Dendritic spine turnover was higher in prospective ArcTom− than in ArcTom+ neurons during baseline, but not after EE (*p = 0.073 and *p* = 0.3399; Mann–Whitney U test corrected for multiple comparisons; *N*_ArcTom−_ = 51, *N*_ArcTom+_ = 48). The turnover of ArcTom− neurons decreased after EE (**p = 0.005 and *p* = 0.8112; Mann–Whitney U test with Bonferroni correction; *N*_ArcTom−_ = 51, *N*_ArcTom+_ = 48). (B) Dendritic spine gain was higher in prospective ArcTom− than in ArcTom+ neurons during baseline, but not after EE (**p = 0.0025 and *p* = 0.123; Mann–Whitney U test corrected for multiple comparisons; *N*_ArcTom−_ = 51, *N*_ArcTom+_ = 48). The gain of ArcTom− neurons decreased after EE. (***p = 0.0001 and *p* = 0.794; Mann–Whitney U test corrected for multiple comparisons; N_ArcTom−_ = 51, *N*_ArcTom+_ = 48). (C) Dendritic spine loss did not differ between prospective ArcTom− and ArcTom+ neurons and was not affected by EE (*p* = 0.089, *p* = 0.425, *p* = 0.034, *p* = 0.876; Mann–Whitney U test with Bonferroni correction; *N*_ArcTom−_ = 51, *N*_ArcTom+_ = 48). (D–F) Baseline dendritic spine turnover, gain, and loss of NexTom− and NexTom+ neurons were not different and were not affected by TAM injection. *p* = 0.7051, *p* = 0.7051, *p* = 0.7526, *p* = 0.132, *p* = 0.3888, *p* = 0.9863, *p* = 0.4146, *p* = 0.0583, *p* = 0.037, *p* = 0.8815, *p* = 0.9314, *p* = 0.7936, *p* = 0.9185; Mann–Whitney U test with Bonferroni correction; *N*_NexTom−_ = 27, *N*_NexTom+_ = 18. (A–F) Boxes are the second and third quartiles and whiskers are the first and last quartiles of the distributions of fractional turnover, gain, and loss of spines per neuron pooled over the epochs reported in the panels. Red: prospective or actual tdTomato+ neurons, gray: prospective or actual tdTomato− neurons. (G) The spine size of ArcTom− and ArcTom+ neurons was stable through time (*p* = 0.635; 2-way ANOVA; *N* = 240) and did not differ between the 2 groups (*p* = 0.34; Mann–Whitney U test; *N* = 240). Each dot represents the measured size of a persistent spine at each imaging time point. Bars are SEM. (H) The dendrite diameter of ArcTom− and ArcTom+ neurons was stable through time (*p* = 0.3176; 2-way ANOVA; *N* = 360) and did not differ between the 2 groups (*p* = 0.6215; 2-way ANOVA; *N* = 360). (A–G) Blue vertical line, time point of EE + TAM injection; gray vertical line, time point of TAM injection. All the data of this figure can be found in the S1 Data file. Arc, activity-regulated cytoplasmic-associated protein; ArcTom, Arc-tdTomato; EE, enriched environment; Nex, neuronal helix-loop-helix protein; NexTom, Nex-tdTomato; TAM, tamoxifen; tdTomato, tandem dimer Tomato.
(TIF)

S3 Fig Transitions and occupancy of recurrent sites. (A, B) Recurrent sites of ArcTom+ and ArcTom− (solid circles, red and black, respectively) and NexTom+ and NexTom− (empty circles, red and black, respectively) neurons were significantly more occupied than expected by chance on most time points. S1 Table for *p*-values. (C–J) Histograms of the number of transitions (C–F) or of time point occupied (G–J) of recurrent synaptic sites of prospective ArcTom+ (full, red), NexTom+ (empty, red), ArcTom− (full, black), and NexTom− (empty, black) neurons during baseline (C, E, G, I) and after EE (D, H) or TAM injection (I, J). Methods for definition of the error bars. All the data of this figure can be found in the S1 Data file. Arc, activity-regulated cytoplasmic-associated protein; ArcTom, Arc-tdTomato; EE, enriched environment; Nex, neuronal helix-loop-helix protein; NexTom, Nex-tdTomato; tdTomato, tandem dimer Tomato.
(TIF)

S4 Fig Correlations of density, survival, and gain rates of dendritic spines of CA1 PNs with freezing in a TFC task. (A–D) The density of spines after EE + TAM injection or after TAM injection of Arc-Cre^ERT2^; Ai9; Thy1-eGFP (solid circles) and Nex-Cre^ERT2^; Ai9; Thy1-eGFP (open circles) mice did not correlate to freezing to the context or to the tone. (E–H) The time constant of the surviving fraction of spines after EE + TAM injection or after TAM injection of Arc-Cre^ERT2^; Ai9; Thy1-eGFP; (solid circles) and Nex-Cre^ERT2^; Ai9; Thy1-eGFP (open circles) mice did not correlate to freezing to the context or to the tone. (I–L) The time constants of the fraction gained of spines after the EE + TAM injection or after TAM injection of Arc-Cre^ERT2^; Ai9; Thy1-eGFP (solid circles) and Nex-Cre^ERT2^; Ai9; Thy1-eGFP (open circles) mice did not correlate to freezing to the context or to the tone. All the data of this figure can be found in the S1 Data file. Arc, activity-regulated cytoplasmic-associated protein; CA1, cornu ammonis 1; EE, enriched environment; eGFP, enhanced green fluorescent protein; ERT2, estrogen receptor triple mutant 2; Nex, neuronal helix-loop-helix protein; PN, pyramidal neuron; TAM, tamoxifen; TFC, trace fear conditioning; Thy1, thymocyte differentiation antigen 1.
(TIF)

S1 Table List of statistical tests, *p*-values, and *N* values sorted by figure. (XLSX)

S2 Table Number of mice, cells, dendrites, and dendritic spines analyzed. (DOCX)

S1 Data Individual quantitative observations that underlie the data summarized in the figures and results of the paper. In Figs 3 and 4 and S3 Fig, to quantify the confidence intervals in the measured fractions of spine gain, spine loss, spine turnover, Occupancy, and Transitions, we used a 2-level bootstrapping method, which accounts both for heterogeneity between neurons and for heterogeneity between spines within a neuron (“Quantification and statistical analysis” in the Methods section for more details). The procedure was repeated 10,000 times, and the error bars denote the standard deviations of the distribution of these quantities across the different samples.
(XLSX)

## Abbreviations

Arc: activity-regulated cytoskeleton-associated protein
ArcTom: Arc-tdTomato
CA1: cornu ammonis 1
CREB: cAMP response element-binding protein
EE: enriched environment
eGFP: enhanced green fluorescent protein
ERT2: estrogen receptor triple mutant 2
GEMM: genetically engineered mouse models
GUI: graphical user interface
HC: home cage
IDF: integrated density of fluorescence
IEG: Immediate Early Gene
LTP: long-term potentiation
Nex: neuronal helix-loop-helix protein
NexTom: Nex-tdTomato
PBS: phosphate-buffered saline
PFA: paraformaldehyde
PN: pyramidal neuron
ROI: region of interest
TAM: tamoxifen
tdTomato: tandem dimer Tomato
TFC: trace fear conditioning
Thy1: thymocyte differentiation antigen 1
TRAP: targeted recombination in active populations
2P: 2-photon
