# Supplementary figures and images for: Hippocampal neurons with stable excitatory connectivity become part of neuronal representations
Source: PLoS Biol. 2020 Nov 3;18(11):e3000928. doi: 10.1371/journal.pbio.3000928 (PMC7665705; doi:10.1371/journal.pbio.3000928)

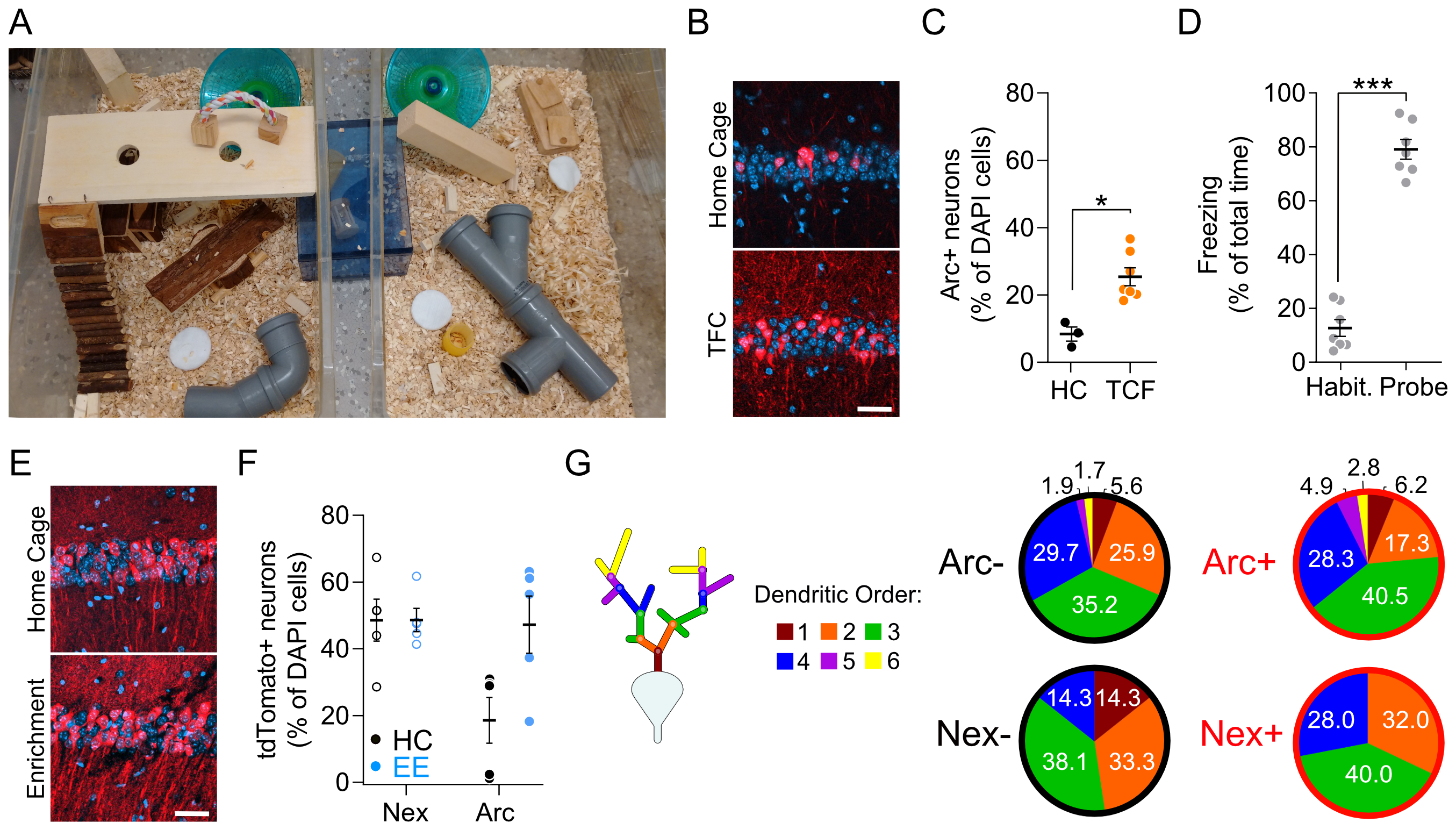

Supplement: S1 Fig — (A) Picture showing a representative EE. (B) Confocal images of the dorsal CA1 of Arc-CreERT2; Ai9 animals after permanence in HC (up) or after a TFC training. Mice received 75 mg/kg TAM. Single z-plane (5-μm z-step, 8–10 focal planes). Scale bar, 100 μm (C) The proportion of ArcTom+ neurons (solid orange circles) after TFC was significantly higher than the proportion of ArcTom+ neurons (solid black circles) after HC. p = 0.0167; Mann–Whitney U test; NHC = 3, NTFC = 6. Horizontal bars: means ± SEM. (D) Arc-CreERT2; Ai9 mice displayed increased freezing during the context probe trial (***p < 0.0006; Mann–Whitney U test; N = 7). Circles are percentage freezing for each mouse during 3 min exposure to the context during training and 3 min exposure to the context during probe. Horizontal bars: means ± SEM. (E) Confocal images of the dorsal CA1 of Nex-CreERT2; Ai9 animals after 2 h permanence in HC (up) or in EE (low). Mice received 75 mg/kg TAM. Single z-plane (5-μm z-step, 8–10 focal planes). Scale bar, 100 μm (F) The proportion of NexTom+ (empty circles) after 75-mg/kg TAM injection was not different between exposure to HC (black) and EE (blue) but different from ArcTom+ neurons (solid circles). p = 0.84; Mann–Whitney U test; N = 5 mice per group. Error bars are SEM. (G) Schematic definition of the dendritic order of the imaged dendrites and proportion of dendrites in each class per group. All the data of this figure can be found in the S1 Data file. Arc, activity-regulated cytoplasmic-associated protein, ArcTom, Arc-tdTomato; CA1, cornu ammonis 1; EE, enriched environment; ERT2, estrogen receptor triple mutant 2; HC, home cage; Nex, neuronal helix-loop-helix protein; NexTom, Nex-tdTomato; TAM, tamoxifen; tdTomato, tandem dimer Tomato. (TIF) [file pbio.3000928.s001.tif]

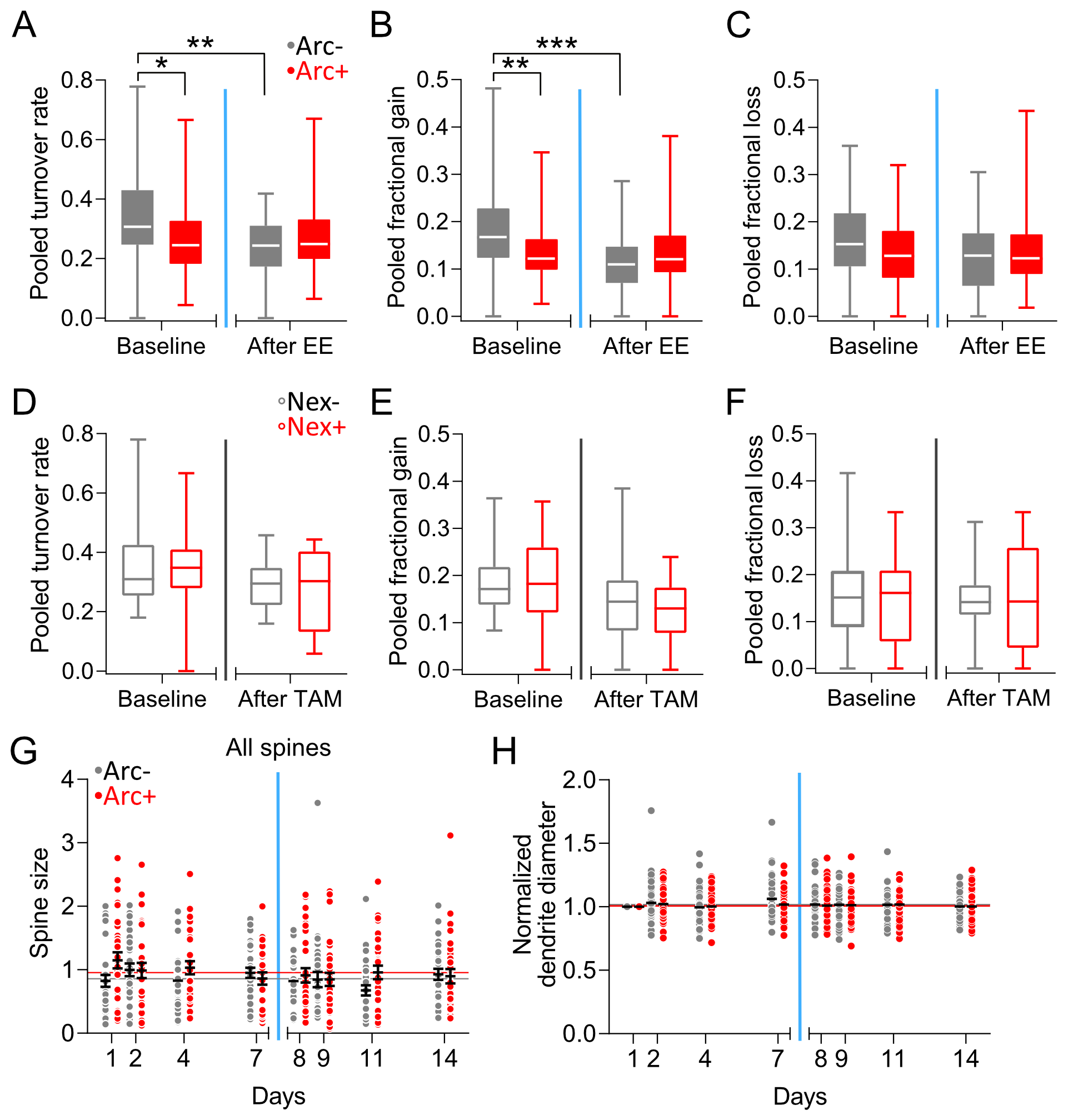

Supplement: S2 Fig — (A) Dendritic spine turnover was higher in prospective ArcTom− than in ArcTom+ neurons during baseline, but not after EE (*p = 0.073 and p = 0.3399; Mann–Whitney U test corrected for multiple comparisons; NArcTom− = 51, NArcTom+ = 48). The turnover of ArcTom− neurons decreased after EE (**p = 0.005 and p = 0.8112; Mann–Whitney U test with Bonferroni correction; NArcTom− = 51, NArcTom+ = 48). (B) Dendritic spine gain was higher in prospective ArcTom− than in ArcTom+ neurons during baseline, but not after EE (**p = 0.0025 and p = 0.123; Mann–Whitney U test corrected for multiple comparisons; NArcTom− = 51, NArcTom+ = 48). The gain of ArcTom− neurons decreased after EE. (***p = 0.0001 and p = 0.794; Mann–Whitney U test corrected for multiple comparisons; NArcTom− = 51, NArcTom+ = 48). (C) Dendritic spine loss did not differ between prospective ArcTom− and ArcTom+ neurons and was not affected by EE (p = 0.089, p = 0.425, p = 0.034, p = 0.876; Mann–Whitney U test with Bonferroni correction; NArcTom− = 51, NArcTom+ = 48). (D–F) Baseline dendritic spine turnover, gain, and loss of NexTom− and NexTom+ neurons were not different and were not affected by TAM injection. p = 0.7051, p = 0.7051, p = 0.7526, p = 0.132, p = 0.3888, p = 0.9863, p = 0.4146, p = 0.0583, p = 0.037, p = 0.8815, p = 0.9314, p = 0.7936, p = 0.9185; Mann–Whitney U test with Bonferroni correction; NNexTom− = 27, NNexTom+ = 18. (A–F) Boxes are the second and third quartiles and whiskers are the first and last quartiles of the distributions of fractional turnover, gain, and loss of spines per neuron pooled over the epochs reported in the panels. Red: prospective or actual tdTomato+ neurons, gray: prospective or actual tdTomato− neurons. (G) The spine size of ArcTom− and ArcTom+ neurons was stable through time (p = 0.635; 2-way ANOVA; N = 240) and did not differ between the 2 groups (p = 0.34; Mann–Whitney U test; N = 240). Each dot represents the measured size of a persistent spine at each imaging time point [file pbio.3000928.s002.tif]

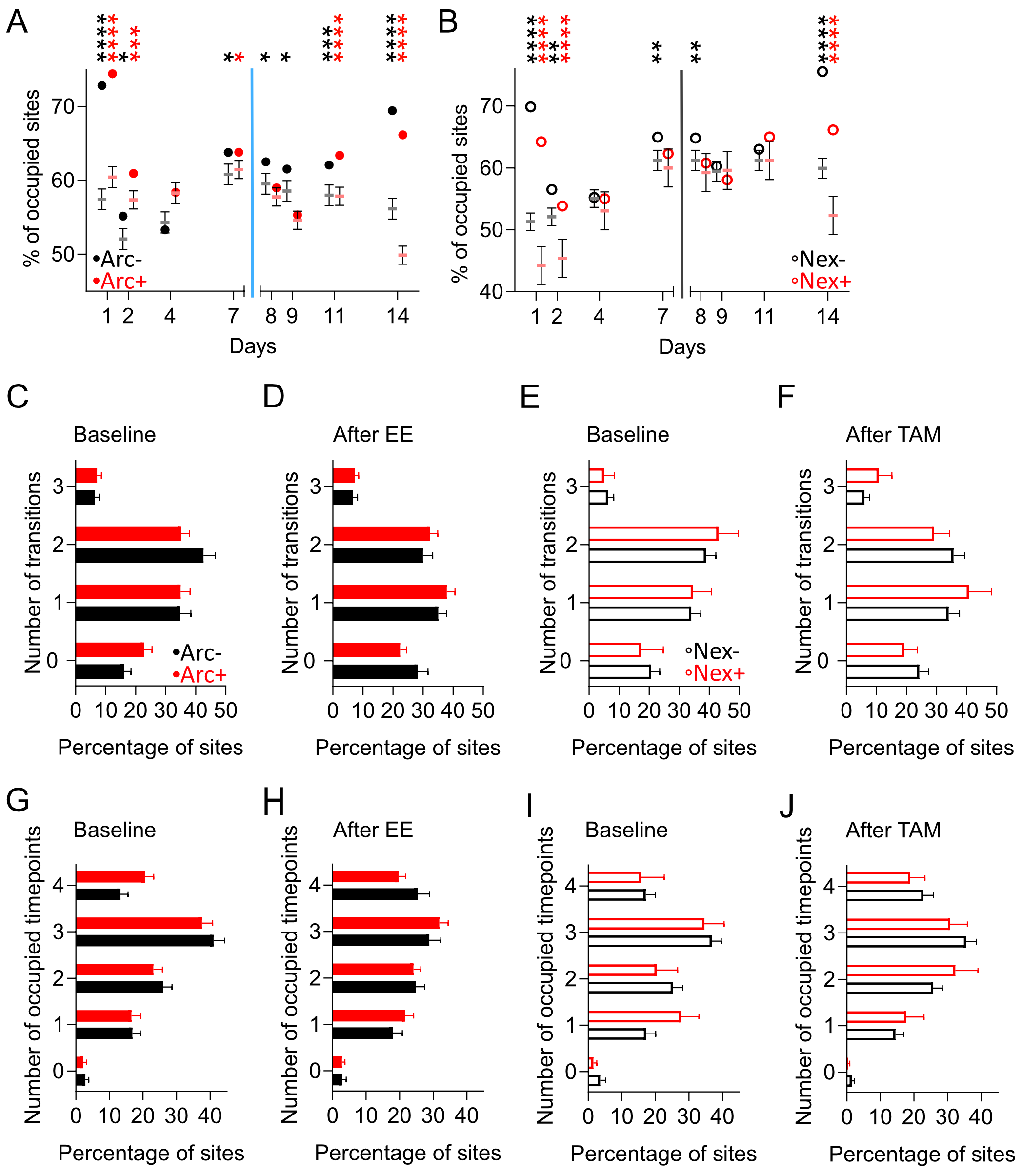

Supplement: S3 Fig — (A, B) Recurrent sites of ArcTom+ and ArcTom− (solid circles, red and black, respectively) and NexTom+ and NexTom− (empty circles, red and black, respectively) neurons were significantly more occupied than expected by chance on most time points. S1 Table for p-values. (C–J) Histograms of the number of transitions (C–F) or of time point occupied (G–J) of recurrent synaptic sites of prospective ArcTom+ (full, red), NexTom+ (empty, red), ArcTom− (full, black), and NexTom− (empty, black) neurons during baseline (C, E, G, I) and after EE (D, H) or TAM injection (I, J). Methods for definition of the error bars. All the data of this figure can be found in the S1 Data file. Arc, activity-regulated cytoplasmic-associated protein; ArcTom, Arc-tdTomato; EE, enriched environment; Nex, neuronal helix-loop-helix protein; NexTom, Nex-tdTomato; tdTomato, tandem dimer Tomato. (TIF) [file pbio.3000928.s003.tif]

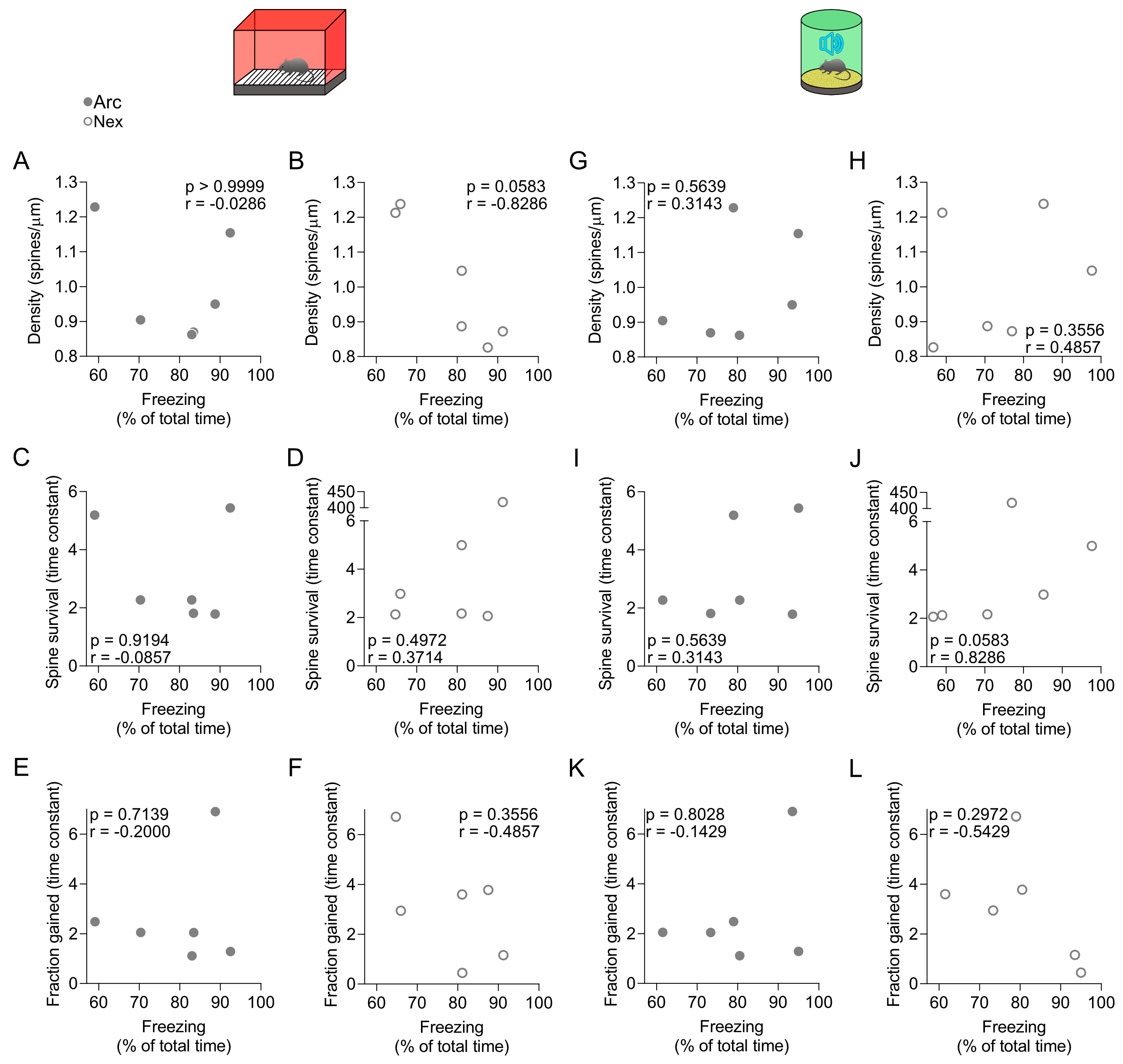

Supplement: S4 Fig — (A–D) The density of spines after EE + TAM injection or after TAM injection of Arc-CreERT2; Ai9; Thy1-eGFP (solid circles) and Nex-CreERT2; Ai9; Thy1-eGFP (open circles) mice did not correlate to freezing to the context or to the tone. (E–H) The time constant of the surviving fraction of spines after EE + TAM injection or after TAM injection of Arc-CreERT2; Ai9; Thy1-eGFP; (solid circles) and Nex-CreERT2; Ai9; Thy1-eGFP (open circles) mice did not correlate to freezing to the context or to the tone. (I–L) The time constants of the fraction gained of spines after the EE + TAM injection or after TAM injection of Arc-CreERT2; Ai9; Thy1-eGFP (solid circles) and Nex-CreERT2; Ai9; Thy1-eGFP (open circles) mice did not correlate to freezing to the context or to the tone. All the data of this figure can be found in the S1 Data file. Arc, activity-regulated cytoplasmic-associated protein; CA1, cornu ammonis 1; EE, enriched environment; eGFP, enhanced green fluorescent protein; ERT2, estrogen receptor triple mutant 2; Nex, neuronal helix-loop-helix protein; PN, pyramidal neuron; TAM, tamoxifen; TFC, trace fear conditioning; Thy1, thymocyte differentiation antigen 1. (TIF) [file pbio.3000928.s004.tif]
