# Supplementary material for: Hippocampal neurons with stable excitatory connectivity become part of neuronal representations
Source: PLoS Biol. 2020 Nov 3;18(11):e3000928. doi: 10.1371/journal.pbio.3000928 (PMC7665705; doi:10.1371/journal.pbio.3000928)
Supplement: S2 Table — (DOCX) [file pbio.3000928.s006.docx]

**S2 Table.**

Number of mice, cells, dendrites and dendritic spines analyzed

|  |  | **Arc-Cre^ERT2^** | |  | **Nex-Cre^ERT2^** | |
| --- | --- | --- | --- | --- | --- | --- |
|  |  | **ArcTom-** | **ArcTom+** |  | **NexTom-** | **NexTom+** |
| **Mice** | Sex | 3 ♂ | 4 ♀ |  | 1 ♂ | 5 ♀ |
|  | *Sum* | *7* | |  | *6* | |
| **Cells imaged** |  | 19 | 25 |  | 10 | 11 |
|  | *Sum* | *44* | |  | *21* | |
| **Cells analyzed** |  | 17 | 16 |  | 9 | 6 |
|  | *Sum* | *33* | |  | *15* | |
| **Dendrites imaged** |  | 77 | 121 |  | 61 | 44 |
|  | *Sum* | *198* | |  | *105* | |
| **Dendrites analyzed** |  | 54 | 81 |  | 42 | 25 |
|  | *Sum* | *135* | |  | *67* | |
| **Spines analyzed** | Mean per day | 1411 | 2240 |  | 1010 | 461 |
|  | Stdv | 31 | 58 |  | 44 | 32 |
|  | Range | 1354 - 1454 | 2113 - 2316 |  | 937 - 1049 | 411 - 495 |
|  | Mean per cell | 83 | 140 |  | 99 | 76 |
|  | Stdv | 90 | 189 |  | 66 | 87 |
|  | Range | 16 - 374 | 17 - 639 |  | 11 - 116 | 9 - 172 |
|  | *Sum* | *11.289* | *17919* |  | *8083* | *3691* |
